# Endovascular treatment of visceral artery and renal aneurysms (VRAA) using a constant mesh density flow diverting stent

**DOI:** 10.1186/s42155-019-0057-1

**Published:** 2019-05-03

**Authors:** Julian Maingard, Anthony Lamanna, Hong Kuan Kok, Dinesh Ranatunga, Rajeev Ravi, Ronil V. Chandra, Michael J. Lee, Duncan Mark Brooks, Hamed Asadi

**Affiliations:** 1 Interventional Radiology Service, Department of Radiology, Austin Hospital, Heidelberg, Melbourne, Victoria 3084 Australia; 2 Interventional Neuroradiology Service, Radiology Department, Austin Hospital, Melbourne, Australia; 3 Faculty of Health, School of Medicine, Deakin University, Waurn Ponds, Australia; 4 Interventional Radiology Service, Department of Radiology, Northern Hospital, Melbourne, Australia; 5 Interventional Neuroradiology Unit, Monash Imaging, Melbourne, Australia; 6 Faculty of Medicine, Nursing and Health Sciences, Monash University, Melbourne, Australia; 7 Interventional Radiology Service, Department of Radiology, Beaumont Hospital, Dublin, Ireland; 8 Royal College of Surgeons in Ireland, Dublin, Ireland; 9 Stroke Division, Florey Institute of Neuroscience and Mental Health, University of Melbourne, Melbourne, Australia

**Keywords:** Visceral artery, Aneurysm, Hepatic artery, Renal artery, Surpass, Flow diverting stent, Endovascular

## Abstract

**Background:**

Flow diverting stents have been used safely and effectively for the treatment of intracranial aneurysms, particularly for large and wide necked aneurysms that are not amenable to conventional endovascular treatment with coiling. The Surpass Streamline device (Stryker Neurovascular, MI, USA) is a relatively new and unique flow diverting stent which maintains constant device mesh density over varying vessel diameters. This may potentially provide advantages compared to other flow diverting stents in achieving aneurysmal occlusion.

**Case presentation:**

Two patients with VRAA were treated using the Surpass Streamline device. The first patient was a 65-year-old male with an incidental 2.4 cm aneurysm originating from the hepatic artery near the gastroduodenal artery (GDA). The second patient was a 56-year-old male with an incidental 1.9 cm renal aneurysm arising from an anterior inferior segmental branch of the left renal artery. A Surpass flow diverting stent was used to successfully exclude the aneurysm neck in both cases.

Reduced flow was achieved in one patient (equivalent to O’Kelly-Marotta [OKM] Grade B1). Preserved flow and stagnation (equivalent to OKM Grade A3) was achieved in the other. There was preserved distal flow in the parent arteries. No immediate complications were encountered in either case. Complete occlusion of both aneurysms was seen on follow up CT angiographic imaging within 8-weeks.

**Conclusions:**

The Surpass flow diverting stent can be used safely and effectively to treat VRAA. It should be considered in unruptured large and giant wide necked VRAAs aneurysms. Additional large prospective studies are required for further validation.

## Background

**Fig. 1:**
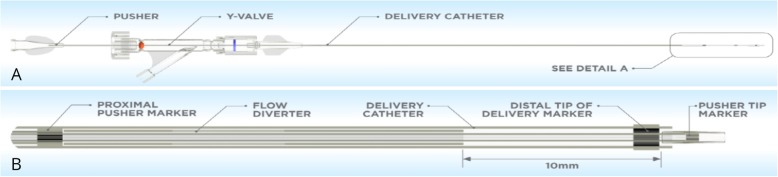
**a**) and **b**) the Surpass Streamline flow diverter delivery system

Flow diversion is a well-established technique for the treatment of intracranial aneurysms with high technical success and low recurrence rates (Cagnazzo et al., 2017; Lv et al., 2016; Lylyk et al., 2009; Madaelil et al., 2017; Pierot et al., 2018; Pumar et al., 2017). The unique properties of flow diverting stents allows for rapid and significant flow reduction within the aneurysm without compromising parent artery flow (Dholakia et al., 2017; Kadirvel et al., 2014). In neurovascular practice, there is a wide armamentarium of available flow diverters including the Pipeline Embolisation Device (PED, Medtronic, MN, USA), SILK flow diverter (Balt Extrusion, Montmorency, France) and the Flow Reduction Endoluminal Device (FRED, Microvention, CA, USA), p64 (Phenox, Bochum, Germany). These devices are effective in the treatment of large or giant complex aneurysms such as those with a wide neck or fusiform appearance. More recently, the Surpass Streamline device (Fig. 1), a self-expandable braided flow diverting stent composed of a cobalt-chromium alloy has been shown to effectively treat such aneurysms with 98% technical success and high occlusion rates at follow up (Wakhloo et al., 2015). It has a significantly lower porosity than currently available flow diverting stents with metal surface area coverage of 30%, a high mesh density and single-layer braided and tubular structure with an increased filament density which facilitates deployment and a braid angle which prevents changes to mesh density as luminal diameter and vascular curvature changes. Increased metal surface area coverage and mesh density assists aneurysm occlusion through increased flow reduction. The device is re-sheathable, and continuous wire access can be maintained tortuous anatomy providing additional advantages in device deployment.

Visceral and renal artery aneurysms (VRAA) are more frequently being detected with the increasing use of cross sectional imaging. They are rare with an estimated prevalence of 2–3% (Hossain et al., 2001) but rupture can be associated with significant morbidity and mortality. Risk factors for VRAA rupture include rapid interval enlargement, size > 2 cm, systemic hypertension, portal hypertension (hepatic and splenic VAA), organ transplantation and pregnancy, necessitating treatment in certain patient groups (Kok et al., 2016; Loffroy et al., 2015). Additional indications for treatment include those of the gastroduodenal artery and pancreaticoduodenal arcade as these are at high risk of rupture (Loffroy et al., 2015).

We report our preliminary and novel experience using the Surpass flow diverter stent in the treatment of VRAA.

## Case presentation

In our institution retrospective reporting of cases are exempted from the Institutional Review Board.

### Patient 1

**Fig. 2:**
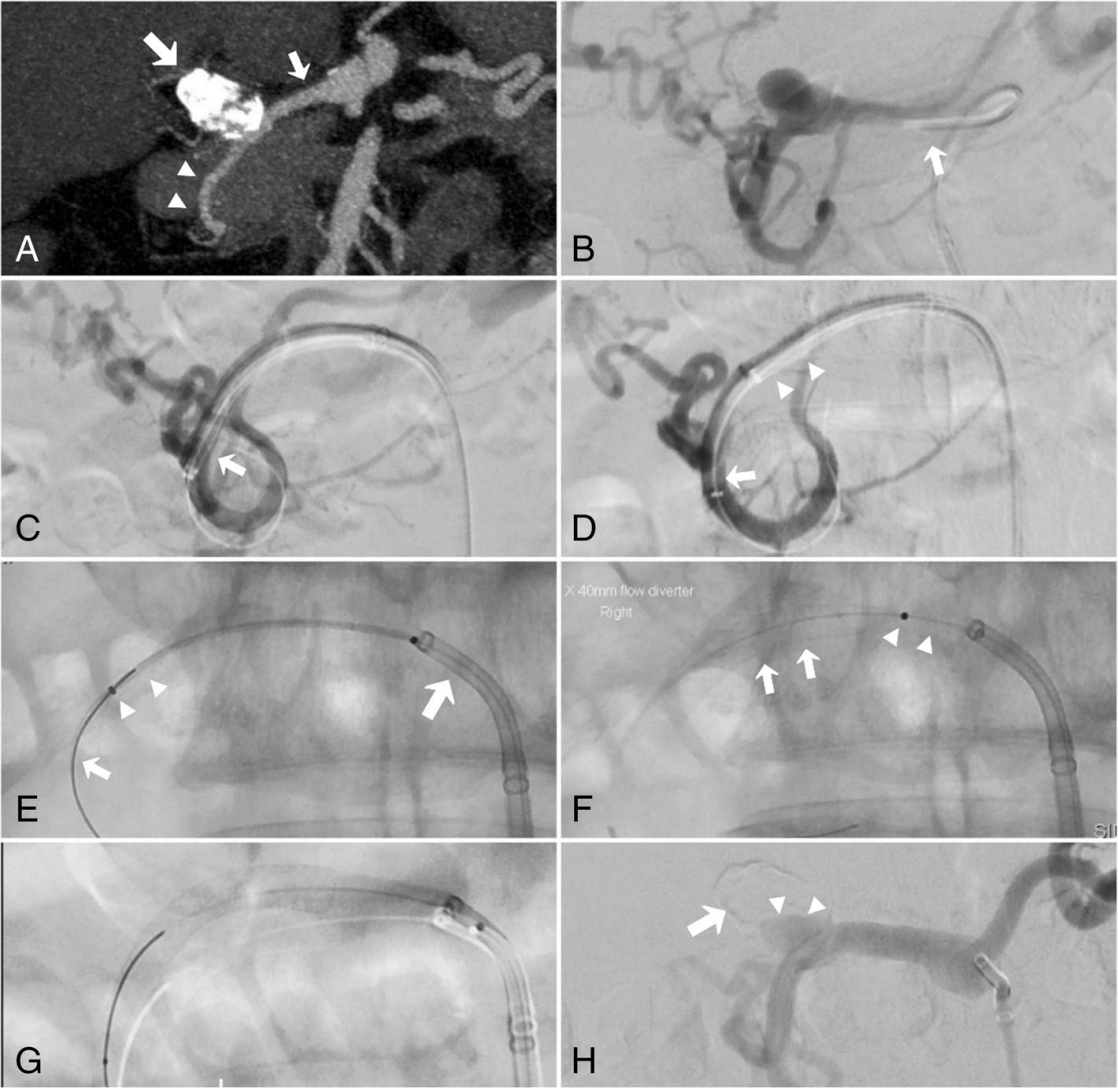
**a**) 2.4 cm Heavily calcified hepatic artery / gastroduodenal artery aneurysm (large arrgow). Common hepatic (small arrow) and gastroduodenal arteries (arrowheads). **b**) Angiography performed via a Cobra-2 catheter (arrow) demonstrating a wide neck unsuitable for conventional coil embolization **c**). An 088 Neuronmax (arrow) was advanced beyond the origin of the aneurysm. **d**) 5Fr Sofia catheter (arrow) acted as the intermediate catheter. The Neuronmax was retracted to the proximal aneurysm neck (arrowheads). **e** to **g**) Retraction of the Surpass delivery system (arrowheads with distal and proximal radio opaque markers) over a wire (arrow in **e**) into the Neuronmax (large arrow) resulting in adequate deployment of the Surpass stent (arrows in **f**). **h**) Final angiogram demonstrates stent patency and reduced aneurysm flow and stagnation (arrowheads). Note the ghost image of the calcified aneurysm rim after subtraction (arrow)

A 65-year-old male presented with an incidental finding of a 2.4 cm partially calcified aneurysm arising at the distal common hepatic artery andinvolving the origin of the gastroduodenal artery (GDA). The size (> 2 cm) and lobulatedmorphology suggested high risk of rupture and so the patient was referred for endovascular treatment. The presence of a wide neck made conventional coil embolization less desirable (Fig. 2a).

Under general anaesthesia, and after 1 week of pre-procedural dual antiplatelet therapy with 100 mg Aspirin and 75 mg clopidogrel, an 8-French introducer sheath (Terumo, Tokyo, Japan) was placed into the right common femoral artery. Initial angiogram of the celiac artery using a 6-French Cobra-2 catheter (Cook Medical, Bloomington, USA) demonstrated a wide-necked aneurysm arising from the terminiation of common hepatic artery involving the origin of the GDA (Fig. 2b). There was aberrant anatomy with hepatic arteries opacified via branches from the GDA and left gastric artery. Subsequently, the Cobra-2 (Cook Medical) was exchanged over a 0.035-in. Amplatz exchange length wire (Cook Medical) for a long 6-French sheath (Neuron MAX 088; Penumbra Inc., CA, USA) which was advanced distal to the aneurysm neck into the gastroduodenal artery (Fig. 2c). Systemic heparin (7000 units) was administered intravenously and a 5-French intermediate catheter (Sofia; MicroVention Inc., CA, USA) was then advanced into the GDA over an 0.035 in. wire and the Neuron MAX sheath was retracted proximal to the aneurysm to a position which maintained stable access (Fig. 2d). A 5 × 40 mm Surpass flow diverting stent (Stryker Neurovascular, MI, USA) was then deployed to cover the aneurysm neck over an 0.014-in. microwire using the push releasing system with good effect (Fig. 2e-g).After the administration of 3 mg intraarterial tirofiban (an antiplatelet agent approved for IV use), the final angiographic image demonstrated reduced flow within the aneurysm (equivalent to O’Kelly-Marotta [OKM] grade B1, Fig. 2h) and preserved flow distally into the GDA and hepatic artery branches. The OKM grading system is summarized in Table 1. Haemostasis was secured with an 8-French vascular closure device (AngioSeal; Terumo). No complications were encountered. The patient was commenced on dual antiplatelet therapy with 100 mg aspirin and 75 mg clopidogrel with a plan to discontinue clopidogrel after 6 months.

**Table 1:** O’Kelly-Marotta grading scale used to assess the degree of angiographic filling and contrast stasis when using flow diverter stents to treat aneurysms

		Stasis Phase		
		1: no stasis (arterial phase clearance prior to capillary phase)	2: moderate stasis (arterial phase clearance prior to venous phase)	3: significant stasis (persistent contrast at venous phase)
Aneurysm Filling	A: total filling (> 95%)	A1	A2	A3
	B: subtotal filling (5–95%)	B1	B2	B3
	C: entry remnant (1–5%)	C1	C2	C3
	D: no filling (0%)	D	N/A	N/A

### Patient 2

A 56-year-old male was referred for treatment of a 1.9 cm renal aneurysm arising from an anterior inferior segmental branch of the left renal artery detected on cross sectional imaging due to interval growth approaching our institutions 2 cm threshold size for treatment.

**Fig. 3:**
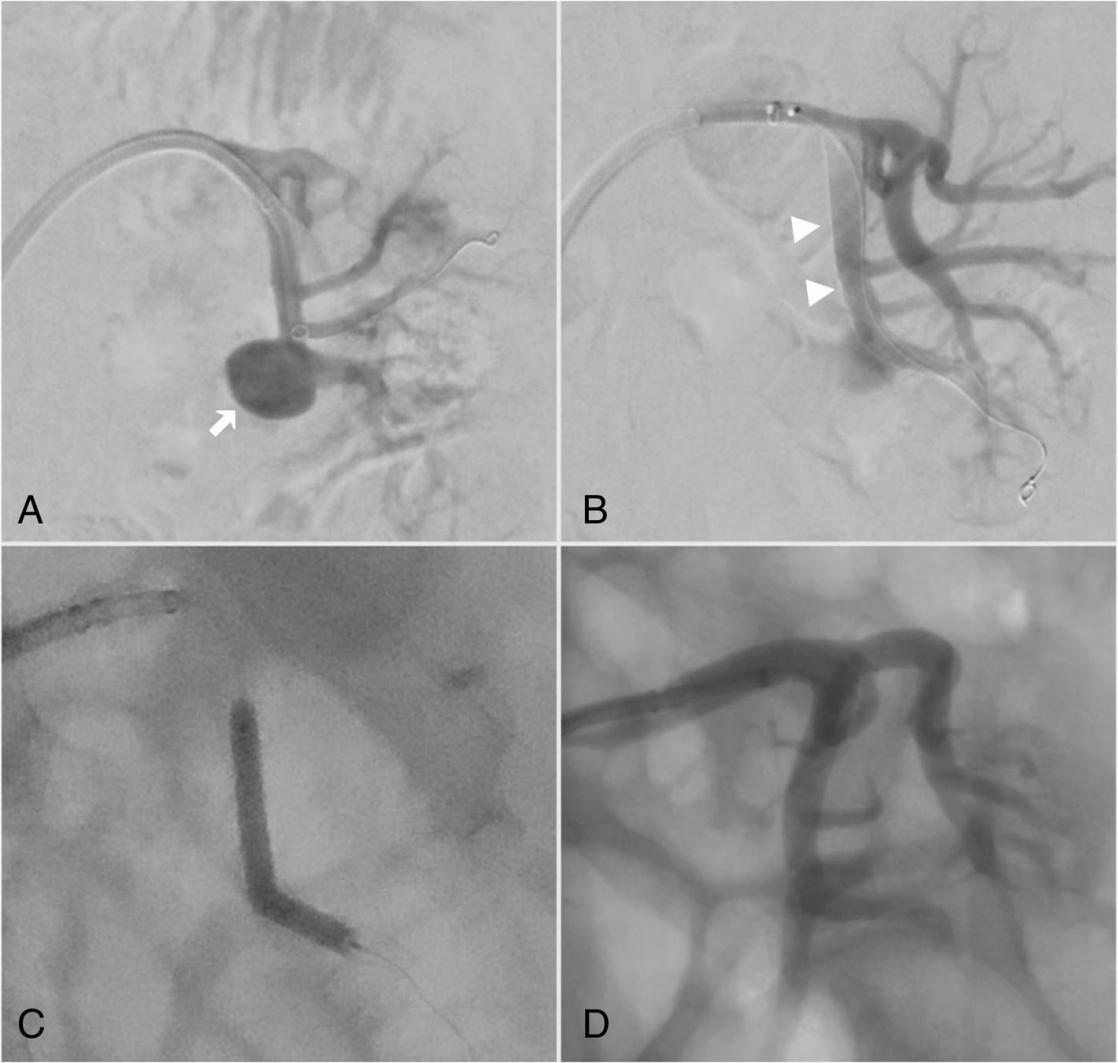
DSA during treatment of a left anterior and inferior segmental renal artery aneurysm. **a**) 1.9 cm wide necked segmental renal artery aneurysm (arrow) felt to be inappropriate for coil embolization. Note the 088 Neuronmax catheter. **b**) Deployed Surpass Streamline flow diverter (arrowheads) with some expected residual aneurysm flow. **c**) a 4 × 20 mm Armada balloon was used to improve stent apposition with the proximal and distal vessel wall landing zones and to ensure exclusion of the aneurysm neck given the origin of the aneurysm from an outer convexity. **d**) post treatment angiogram with minimal aneurysm flow and preserved end organ perfusion

Under general anaesthesia, and after 1 week of pre-procedural dual antiplatelet therapy with 100 mg Aspirin and 75 mg clopidogrel, an 8-French introducer sheath (Terumo) was placed into the right common femoral artery. Angiography via a 6-French Cobra-2 catheter (Cook Medical) demonstrated a 1.9 cm wide-necked renal artery aneurysm arising from an anterior inferior segmental branch of the left renal artery (Fig. 3a). Systemic heparin (8000 units) was administered intravenously. A long 6-French introducer sheath (Neuron MAX 088; Penumbra) was then advanced into the proximal segment of the renal artery. A 1.7-French microcatheter (Excelsior SL 10 45°; Stryker Neurovascular) was then navigated past the aneurysm neck. A 5x50mm Surpass flow diverter stent (Stryker Neurovascular) was then deployed across the aneurysm neck (Fig. 3b), push, OTW, 0.014 wire. Good wall apposition was achieved after balloon expansion using a 4 × 20 mm non-compliant angioplasty balloon (Armada 14; Abbott Vascular, Chicago, IL, USA) (Fig. 3c). After 3 mg of intraarterial tirofiban, completion angiography demonstrated successful stagnation of flow within the aneurysm (equivalent to OKM grade A3, Fig. 3d) and preservation of flow into the inferior pole branch. Haemostasis was achieved with a vascular closure device (AngioSeal; Terumo). No complications were encountered. The patient was commenced on dual antiplatelet therapy with 100 mg aspirin and 75 mg clopidogrel for 6 months post-procedure.

**Fig. 4:**
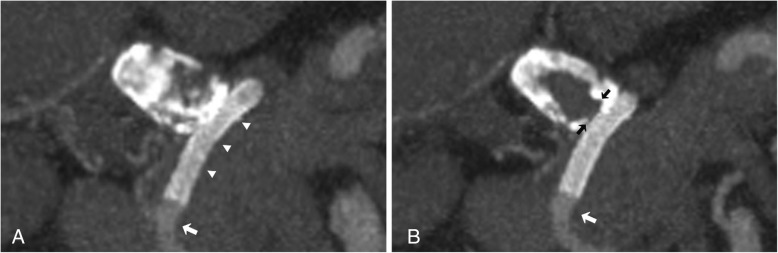
8-week CTA follow up. **a**) and **b**) Coronal maximum intensity projection (MIP) CTA demonstrating patency of the Surpass Streamline flow diverting stent (arrowheads) and preservation of flow into the distal gastroduodenal artery (arrow). Note complete thrombosis of the aneurysm with no residual neck (black arrows)

**Fig. 5:**
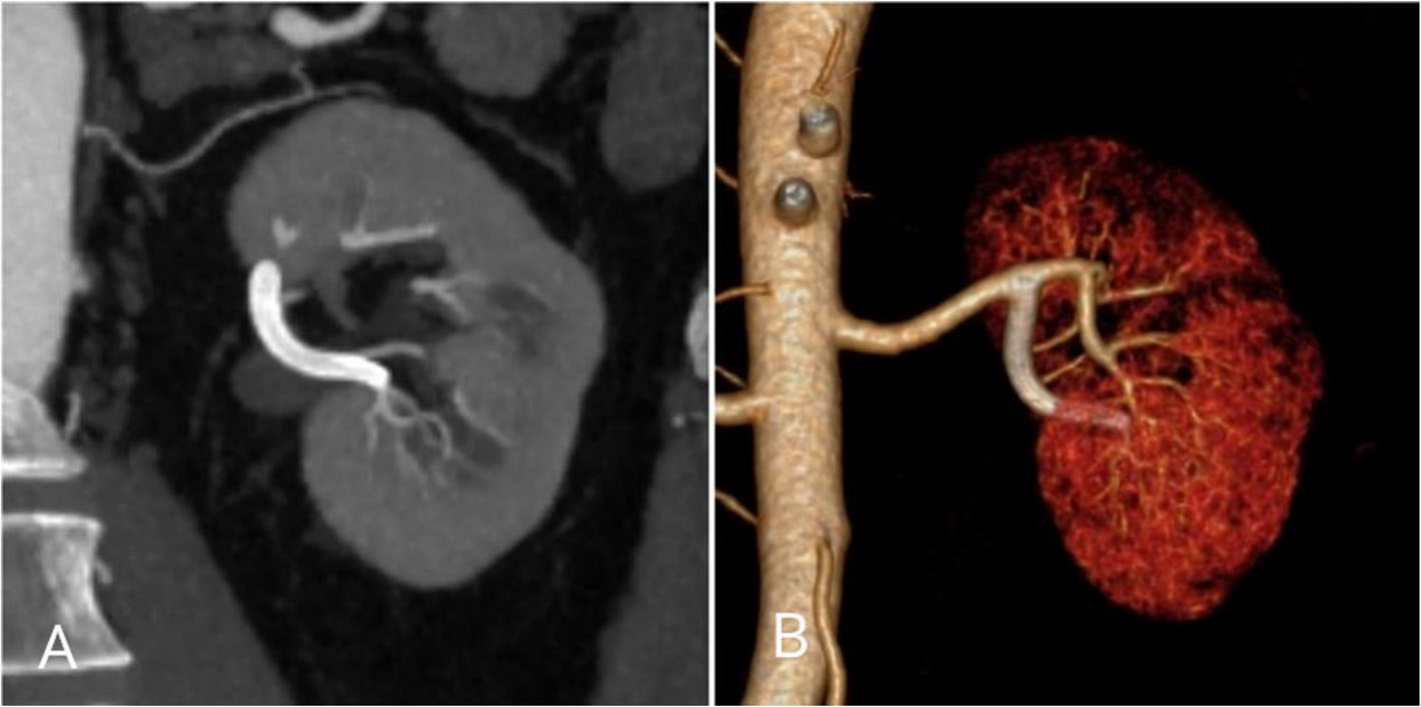
3-week follow up CTA. **a**) coronal MIP CTA and corresponding 3D volume rendered imaging demonstrating complete thrombosis and occlusion of the aneurysm without end organ or stent complication

Both procedures were uncomplicated and technically successful with no immediate or short-term complication. At 8 and 3 weeks follow up CTA imaging respectively both aneurysms were completely occluded (Figs. 4 and 5). Dual antiplatelet therapy will continue for 6 months.

## Discussion

The use of endovascular procedures as first line treatment for the management of VRAA is becoming increasingly popular (Belli et al., 2012). A minimally invasive approach may reduce treatment-related morbidity and mortality with studies demonstrating success comparable to conventional open repair (Kok et al., 2016). Technical success rates as high as 94% with visceral preservation rates up to 99% have been reported. Importantly, there is low treatment-related morbidity (< 4%) (Kok et al., 2016). While the natural history of unruptured VRAA is unclear, rupture is a significant complication and associated with high morbidity and mortality (Belli et al., 2012). Indications for treatment include rapid interval enlargement, size > 2 cm, systemic hypertension, portal hypertension (hepatic and splenic VAA), organ transplantation and pregnancy and true aneurysms of the gastroduodenal artery and pancreaticoduodenal arcade as these are at high risk of rupture (Loffroy et al., 2015).

Conventional endovascular approaches involve selective catheterization of the parent artery and subsequent unassisted coil embolization, allowing for rapid thrombosis of the aneurysm and exclusion from the circulation. Additional endovascular approaches include the use of vascular plugs, front and back door parent artery embolization (where possible), liquid embolic agents such as n-butyl cyanoacrylate or ethylene vinyl copolymer (Onyx) and precipitating hydrophobic injectable liquid (PHIL) (Kok et al., 2016). Parent artery sacrifice is also possible but is associated with the complications of end organ infarction and is not appropriate in many patients.

More recently, covered stent grafts have been used to treat VRAA with a technical success rate of up to 96% and low complications rates reported (Cappucci et al., 2017; Venturini et al., 2017). The ability to cover the aneurysm neck while maintaining flow within the parent artery makes these devices useful when the target aneurysm has a large neck or unfavorable morphology for coiling. They are particularly useful in the treatment of pseudoaneurysms. However, covered stent grafts are limited by their physical properties including higher radial opening force and increased rigidity. As a result, they often force the parent artery to adapt to their shape resulting in loss of the natural arterial curvilinear course with the potential for end organ thromboembolic complications. Additionally, covered stents obstruct side branch perfusion and have a large profile making their use in smaller vessels potentially more difficult.

Recently, neurointerventional approaches used commonly in the treatment of intracranial aneurysms have found application in the peripheral vasculature and for VRAA (Maingard et al., 2017). Balloon-assisted and stent-assisted approaches as well as novel scaffolding devices have made it possible to treat complex aneurysms with unfavourable morphology safely and effectively (Marotta et al., 2018; Spiotta et al., 2016; Labeyrie et al., 2017; Lubicz et al., 2016). Complete occlusion has been demonstrated at short and mid-term follow up (Pierot et al., 2018; Wakhloo et al., 2015; Maingard et al., 2017; Labeyrie et al., 2017; Lubicz et al., 2016). Ideal characteristics of a stent include low profile, flexibility and adaptability to varying parent vessel diameters and aneurysm lengths.

Flow diversion for intracranial aneurysms is a well-established approach particularly in those with complex or difficult morphology or location (Rajah et al., 2017). These devices allow parent artery remodeling while significantly reducing aneurysmal flow leading to eventual thrombosis. Computational flow dynamic models of commercially available flow diverters have demonstrated reductions in aneurysmal flow between 65% and 82%. Increased porosity and reduced pore density are associated with further reductions in aneurysm flow (Dholakia et al., 2017).

Several well-known and FDA approved endoluminal devices are available including the Pipeline Embolisation Device (PED, Medtronic, MN, US), Silk (Balt Extrusion, Montmorency, France), Flow Reduction Endoluminal Device (FRED, MicroVention Inc., CA, USA), p64 (Phenox, Bochum, Germany) and LEO Baby (Balt Extrusion, Montmorency, France). The unique braided design of these devices produces low radial opening forces to facilitate navigation while greater metal coverage and reduced porosity encourages aneurysm occlusion. Rapid flow reduction promotes thrombosis within hours to days, and sometimes months, and provides a scaffold for neointimal proliferation over the aneurysm neck (Dholakia et al., 2017; Kadirvel et al., 2014). The braided design and metallic coverage disrupts in- and outflow patterns at the aneurysm neck causing sluggish flow and increased blood viscosity, promoting thrombosis, while perforator and side branch perfusion is preserved. These devices have proven efficacy and safety for the treatment of a wide range of intracranial aneurysms (Madaelil et al., 2017; Bhogal et al., 2017; Zanaty et al., 2014; Zhou et al., 2017). These unique properties have been utilized when treating peripheral vasculature pathology (including VRAA) with good technical success (Sfyroeras et al., 2012).

More recently the Surpass Streamline flow diverting stent has achieved FDA approval for investigational use in the intracranial vasculature with preliminary results demonstrating high safety and efficacy. Large series demonstrate complete aneurysm occlusion comparable to stent-assisted coil embolization (up to 94%) with no reported mortalities (Wakhloo et al., 2015; De Vries et al., 2013). Large trials are ongoing (Kan et al., 2016).

The stent is a self-expanding tubular mesh composed of cobalt chromium. It has a significantly lower porosity than currently available flow diverting stents with a metal surface area coverage of 30%, a high mesh density and single-layer braided and tubular structure. The device comes in varying diameters including 3, 4 and 5 mm and lengths ranging from 12 to 50 mm and is deployed via a 3.7 French distal catheter with a pusher. While the increased filament density facilitates deployment and a braid angle prevents changes to mesh density as luminal diameter and vascular curvature changes, an increased metal surface area coverage and mesh density assists aneurysm occlusion with an added 18–22% reduction in aneurysm flow. Platinum wires with the braided mesh design also improves visualization during deployment. The delivery system tracks over any 0.014 in. wire allowing more distal and smaller vessel deployment.

In both cases, the wide necked morphology, parent vessel diameter and curvature (both aneurysm arising from the outer convexity) and the presence of important arterial branches precluded the use of conventional coil embolization, standard self-expanding or balloon-expandable covered stents. The Surpass flow diverter was ideally suited obviating the risk of distal end organ ischaemic complications. Recent publications demonstrate successful treatment of visceral aneurysms with stents with flow diverting properties, where similarly, parent and side branch patency is deemed important. The double-layer micromesh Roadsaver, similar to the Surpass, has increased mesh density and reduced poor size resulting in improved aneurysm occlusion (Akkan et al., 2017).

Importantly, after deployment of a flow diverting stent persistent flow within an aneurysm is expected immediately post procedure. In contradistinction to covered stent grafts, the Surpass flow diverter porosity and pore density reduces but does not completely occlude aneurysm flow. On the final angiogram, reduced but persistent flow was observed in patient 1 (OKM Grade B1) indicating successful treatment. Importantly, in patient 2 no change in flow was observed. However, there was stagnation of contrast within the aneurysm (OKM Grade A3). The reduction in flow and stagnation highlights the advantage of the Surpass flow diverter which allows for preservation of side branch and perforator vessels. It is important for operators using this device to be aware that it can take hours to days to achieve complete aneurysm occlusion. At 8 week and 3 weeks respectively both aneurysms were completely occluded on follow up imaging. Systemic anticoagulation and antiplatelet agents may interfere with this; however, a short course of dual antiplatelet therapy is required to maintain stent patency until endothelialization occurs.

We advocate for the use of dual antiplatelet therapy to prevent in-stent thrombosis when using flow diverting stents for peripheral aneurysms. Both patients were administered dual antiplatelet therapy for 1 week prior to the procedure (100 mg Aspirin and 75 mg clopidogrel) with the plan to continue this for 6 months postprocedure, at which point clopidogrel is ceased. Most studies evaluating flow diverting stents have used similar regimes and premature cessation of clopidogrel has been linked with a higher risk stent thrombosis, even at 3 months (De Vries et al., 2013). An alternative regime is to load the patients with higher doses of aspirin and clopidogrel in the hours prior to the procedure (Lv et al., 2016).

We encountered no complications in the treatment of our patients. Foreseeable complications include rapid in-stent thrombosis which can be attenuated with the use of systemic heparin and administration of intraarterial antiplatelet agents (e.g. tirofiban) immediately following stent deployment. Importantly, the Surpass flow diverter is limited to use in smaller vessels with a maximum vessel diameter of 5.3 mm with the 5 mm device. Consideration should also be given to material costs compared to conventional endovascular methods.

## Conclusion

The Surpass flow diverting stent demonstrates early safety and efficacy in the treatment of VRAA. This novel report of its use in the peripheral vasculature highlights its expanding role extra-cranially. It should be considered in those aneurysms with wide necks or arising from difficult parent vessel anatomy with the constant mesh density potentially providing improved flow reduction over a range of vessel diameters when compared to other commercially available flow diverting stents. Its use should be further validated in larger case series or prospective studies. There is scope for future work to evaluate the long-term clinical outcomes with this device.

## Abbreviations

CTA: Computed Tomography Angiography
FRED: Flow Reduction Endoluminal Device
GDA: Gastroduodenal artery
OKM: O’Kelly-Marotta
PED: Pipeline Embolisation Device
PHIL: Precipitating Hydrophobic Injectable Liquid
VRAA: Visceral and renal artery aneurysms
